# Challenges in delivery of tuberculosis Services in Ethiopian Pastoralist Settings: clues for reforming service models and organizational structures

**DOI:** 10.1186/s12913-021-06662-3

**Published:** 2021-06-30

**Authors:** Fentabil Getnet, Meaza Demissie, Alemayehu Worku, Tesfaye Gobena, Rea Tschopp, Alinoor Mohamed Farah, Berhanu Seyoum

**Affiliations:** 1 School of Public Health, Jigjiga University, Jigjiga, Ethiopia; 2 School of Public Health, Haramaya University, Dire Dawa, Ethiopia; 3 Addis Continental Institute of Public Health, Addis Ababa, Ethiopia; 4 School of Public Health, Addis Ababa University, Addis Ababa, Ethiopia; 5 Armauer Hansen Research Institute, Addis Ababa, Ethiopia; 6 Swiss Tropical and Public Health Institute, Basel, Switzerland; 7 University of Basel, Basel, Switzerland

**Keywords:** Barriers, Challenges, Tuberculosis, Pastoralist, Somali, Ethiopia

## Abstract

**Background:**

The End-TB strategy aims to see a world free of tuberculosis (TB) by the coming decade through detecting and treating all cases irrespective of socioeconomic inequalities. However, case detections and treatment outcomes have not been as they should be in Somali pastoral settings of Ethiopia. Hence, this study aimed to explore the challenges that hinder the delivery and utilization of TB services in pastoral areas.

**Methods:**

A qualitative study was conducted between December 2017 and October 2018 among pastoralist patients with delay of ≥2 months in seeking healthcare, healthcare providers and programme managers. Data were collected from different sources using 41 in-depth interviews, observations of facilities and a review meeting of providers from 50 health facilities. The data were transcribed, coded and analyzed to identify pre-defined and emerging sub-themes. ATLAS.ti version 7.0 was used for coding data, categorizing codes, and visualizing networks.

**Results:**

Poor knowledge of TB and its services, limited accessibility (unreachability, unavailability and unacceptability), pastoralism, and initial healthcare-seeking at informal drug vendors that provide improper medications were the key barriers hindering the uptake of TB medical services. Inadequate infrastructure, shortage of trained and enthused providers, interruptions of drugs and laboratory supplies, scarce equipment, programme management gaps, lack of tailored approach, low private engagement, and cross-border movement were the major challenges affecting the provision of TB services for pastoral communities. The root factors were limited potential healthcare coverage, lack of zonal and district TB units, mobility and drought, strategy and funding gaps, and poor development infrastructure.

**Conclusion:**

In pastoral settings of Ethiopia, the major challenges of TB services are limited access, illicit medication practices, inadequate resources, structural deficits, and lack of tailored approaches. Hence, for the pastoral TB control to be successful, mobile screening and treatment modalities and engaging rural drug vendors will be instrumental in enhancing case findings and treatment compliance; whereas, service expansion and management decentralization will be essential to create responsive structures for overcoming challenges.

**Supplementary Information:**

The online version contains supplementary material available at 10.1186/s12913-021-06662-3.

## Introduction

Tuberculosis (TB) has remained the top killer infectious disease. Globally, it caused an estimated 10 million illnesses and nearly 1.4 million deaths in 2019. The same year, Ethiopia had an estimated 157,000 TB illnesses, which ranks the country 9th among the 30 high burden countries globally, 3rd in Africa, and among a few countries with the triple burden of TB, MDR-TB and TB/HIV [1]. The highest prevalence of TB was reported among pastoral communities in Ethiopia [2]. To curb the epidemic of the disease, the global End-TB Strategy sets early diagnosis and prompt treatment of all cases as principal components to reduce TB deaths, prevent drug resistance, stop transmission, and ultimately end TB epidemics by 2030 [3]. The ambitious targets can only be realized through the universal provision of biomedical services regardless of socioeconomic disparities, which requires implementing social and economic interventions, universal healthcare coverage, and collaborative efforts from health and other sectors [4].

However, globally, TB biomedical services remain underutilized as over 3 million cases are still missed and optimal treatment outcomes have not been achieved yet [1]. The problem is disproportionately severe in resource-limited settings despite the highest disease toll. The passive case finding strategy left millions of infectious cases undetected in communities [5], and the Directly Observed Treatment-Short Course (DOTS) has not guaranteed optimal treatment success rates [6]. Similarly, around a third of TB cases were not diagnosed and treated in Ethiopia in 2018 [1]. Pieces of evidence indicated that the rates of undiagnosed and untreated cases in certain geographical and socioeconomic settings of the country could be much higher than the aggregate national reports [7, 8]. In Somali Regional State of Ethiopia (SRS), the TB control programme failed to detect and treat around half of the estimated cases in the past years [9], and treatment success rates were reported to be unsatisfactory [10].

The underutilization of biomedical services, monitored by case detections and treatment success rates, could be deep-rooted to a complex patient, societal, and health system causes. The slow progress in service utilization in affected resource-limited settings could have resulted from poor uptake of services by diseased people which is expressed in deprived healthcare-seeking behavior and compliance to services [11], or due to impediments of the National Tuberculosis control Programmes (NTPs) [12]. The performance of NTPs to achieve TB control targets depends on strong health systems and tailored TB control approaches integrated into health systems [13]. However, the health systems in resource-limited countries struggle to attain universal health coverage enabling effective TB diagnostic and treatment services due to gaps in financing, leadership, strategies and policies, resources, and socio-political challenges [14].

The bottlenecks for TB control programme performance could vary in different geopolitical, epidemiological, socioeconomic, and time contexts [15–17]. Pastoralists are among communities disproportionately affected by TB and poor health service coverage [18, 19]. The delivery of healthcare services to pastoralists is difficult because of their seasonal movements following the evolution of climatic conditions and availability of resources, and scattered settlement in broader geographical areas [19, 20]. The fact that pastoralists in the study area have extended families wholly sheltered in petite dome-shaped huts built for transitory purposes creates a favorable environment for transmission. Despite the high vulnerability and TB burden, TB control efficiency metrics have not been satisfactory compared to settled communities in Ethiopia [21, 22]. Moreover, the novel health extension programme, on which the NTP of Ethiopia counts on, has not been effective in identifying presumptive cases in rural households and referring to healthcare units [23].

Due to these reasons, a holistic approach to investigate the TB control programme impediments is essential in proposing context-tailored interventions that would better tackle the socioeconomic and health system determinants of TB services in affected and underserved pastoral communities [24]. Therefore, this study aimed to explore the challenges of TB services utilization and provision for pastoralists through the perspectives of patients who sought delayed healthcare, healthcare providers and programme implementers in Somali Regional State, Ethiopia. It further suggests thoughts and potential solutions for overcoming the challenges to achieve TB control targets in pastoralist settings.

## Methods

### Study setting and period

The study was conducted from December 2017 to October 2018 in Somali Region, Ethiopia. Geographically, the region shares international borders with Kenya to the south, Somalia to the southeast and east, and Djibouti to the northwest. The region contains 11 administrative zones subdivided into 96 districts (*Woredas*), and 6 town councils [25] and is a home of an estimated 5,899,000 inhabitants (3,165,000 male and 2,734,000 female) in 2018 [26]. More than 85% of the population practice pastoralism, either nomadic or agro-pastoralism. The nomadic pastoralists rear livestock and are seasonally migratory, and agro-pastoralists undertake mixed herding and farming forms of subsistence [17, 27]. Regarding health-system organization, the top hierarchy is the Regional Health Bureau (RHB) that manages Woreda/district Health Offices (WoHO) and hospitals. The WoHO, in turn, manages health centers and health posts in each district. In 2018, the region had 10 hospitals and 200 health centers [9]. The hospitals and selected health centers provide TB diagnosis and treatment services according to the national Directly Observed Treatment-Short course (DOTs) strategy [28]. The standard methods for PTB diagnosis include microscopy, Xpert MTB/RIF, chest radiographs, and pathological and clinical investigations. This study was conducted at all TB control programme units; RHB, WoHO (*Adadle, Sheygosh, Dege-habour, and Harshin*), hospitals (*Kharamara, Dege-habour, Kebri-daher, and Gode*), and health centers (*Abilelie, Bohol, Denan, Harshin, and Sheygosh*). The study sites were purposively selected considering geographical representation and high pastoralist habitants, and high TB patient flow was also considered for selection of health facilities.

### Study design

A qualitative study with a phenomenological approach, a useful design to describe the meanings and consequences of experiences [29], was used to provide deeper insights into the perceptions and experiences of delayed pastoralist patients with pulmonary TB on what influenced their healthcare-seeking behavior and compliance to TB services, and the challenges the health system is facing in the provision of TB services for pastoralists. The consolidated criteria for reporting qualitative research (COREQ) checklist was followed to report the important aspects of the study [30].

### Study participants and sampling

Pastoralist patients with newly diagnosed pulmonary TB (PTB) and TB programme implementers were targeted for this study. We purposively selected patients aged ≥18 years who delay 2 months or more in seeking healthcare after the onset of TB symptoms to get rich information on the real and perceived barriers that hindered patients from timely healthcare seeking and impelled them to informal remedial practices. TB programme implementers, whereas, were selected to explore the entire image of the challenges faced by the health system while providing TB services to pastoralists. We included implementers at all levels of the TB control programme including RHB- and WoHO- level managers, DOTs and laboratory providers. We applied purposive selection using deviant and intensive sampling techniques to recruit patients and implementers, respectively [31]. Eligible patients were identified using a screening questionnaire. As newly diagnosed PTB patients arrived at DOTs clinics to initiate treatment, the DOTs providers consecutively interviewed patients to collect data on socio-demographics, symptoms they had, dates of each symptom onset, and date of first provider consultation while they had the sore illness and fresh memory. The duration of delay was then calculated between cough onset (if no cough, symptoms that compelled patients to seek medical attention) and provider consultation. Treatment providers were oriented to immediately inform the investigators before the provision of health education as they met a patient fulfilling the inclusion criteria. Regarding the implementers, we interviewed focal persons of the TB control programme in the selected offices and health facilities.

Determining adequate sample size in qualitative research is a matter of judgment and information saturation and homogeneity of the sample populations [32]. Pastoralists in Somali region have the same ethnicity, culture, religion, language, and lifestyle. Thus, we interviewed 19 pastoralist PTB patients with delay of 2 months or longer (9 female and 10 male). Similarly, all the TB programme implementers were health professionals, so we interviewed 22 respondents including 6 managers (2 RHB and 4 WoHO) and 16 providers at hospitals and health centers (11 DOTs and 5 laboratory providers). The interviews were stopped when we were not able to explore new information from the last 2 recruits. .

### Data collection

Data were collected using different methods and sources including in-depth interviews, facility observations, field track records, and attendance of an annual TB review meeting.

#### In-depth interviews

We conducted face-to-face in-depth interviews with all respondent types using open-ended and semi-thematic guides that were translated into Somali and Amharic languages. The patient interview guide was prepared to get better insights into the patients’ knowledge of TB and its services, perception of illness, reasons for delayed care-seeking, remedies attempted before visiting health facilities, and the barriers to access services. Interview guides for managers, DOTs and laboratory providers were prepared to get deeper insights into the challenges that impede their service provision efforts for pastoralists including factors related to management structure, logistics and supply, human resource, infrastructure, and societal aspects. Healthcare providers were also interviewed about the common myths/misconceptions, informal remedies, and main factors for delayed healthcareseeking in the pastoralist communities (Supplementary file).

Qualitative researchers should entail observing, joining and talking to people in their language and context to understand the phenomenon from their perspectives [32]. The co-author (AM) and a research assistant (MPH holder) who are native speakers of Somali language and integral members of the community conducted all the patient interviews. The interviews were conducted at ventilated places inside the health facilities but away from providers to help them talk freely about their perspectives on TB diagnosis and treatment services. The interviews were conducted in the mornings when patients came to take drugs. Also, the first author (FG) and the research assistant conducted interviews with implementers using Amharic and Somali language depending on respondent preferences, respectively.,. Places and time for interviews were also adjusted based on their preferences. All the interviews were audiotape recorded.

#### Facility observation

TB clinics and laboratories in the study hospitals and health centers were observed using an inventory checklist prepared to assess the availability and readiness of basic resources such as rooms (adequacy and site), patient waiting areas, furniture, water, electricity, medical supplies, equipment and trained staff.

#### Field track record

FG was coordinating the fieldwork of a related facility-based quantitative study between December 2017 and October 2018. During field supervisions, FG collected regular reports using activity tracking sheet on the challenges faced by TB service delivery points including supply, infrastructure, human resource and patient-related challenges. The data collected throughout the year were included in this analysis.

#### Attendance of annual review meeting

The TB control programme office at RHB organized the annual review meeting for 2 days in October 2018 to evaluate the performance of TB services in health facilities. The DOTs providers representing over 50 health facilities presented their performance and challenge reports. FG joined the meeting and took notes on the challenges.

### Data analysis

Verbatim transcriptions were done for audio records.. The first author (FG) transcribed interviews conducted in Amharic language while the co-author (AM) and the assistant researcher transcribed the interviews in Somali language. FG instantly read the translated transcripts to validate the content and quality of data, and corrective measures were taken for any inconveniences as necessary.

Transcripts in *Rich Text Formats* were imported into ATLAS.ti software version 7.0 to produce codes, categorize codes, and visualize networks. FG iteratively read the transcripts to familiarize the contents, context and meanings, and to produce meaningful codes with code meanings. A codebook was produced to identify codes with duplicate meanings followed by code renaming to identify the final list of codes. The codebook was validated by all authors. A framework analysis approach was followed to summarize data and interpret findings. Codes were summarized into categories, categories into pre-defined and emerging subthemes, and finally into two major themes; factors affecting TB services utilization, and factors affecting effective service delivery. The reports have been presented in textual descriptions with selected quotations. Data from observations and reports were analyzed manually and integrated into the results.

### Quality control and trustworthiness

We applied various quality measures to ensure trustworthiness in the findings. The use of different data sources, data collection approaches, and involvement of all contingents in the TB control programme helped to get a holistic understanding, and triangulate statements from the service provider and patient perspectives. Tools and procedures were evaluated before the data collection. The data collection was conducted over a relaxed period, which provided adequate time for transcription and immediate appraisal of data while the data collection was ongoing. Besides deepening the data, field and review meeting observations helped to triangulate information from interviews with programme implementers. All authors participated in the conception, design, development of field guides, and substantiated all processes of the analysis and interpretation of findings.

The prolonged engagement of researchers in the setting was very helpful to a better understanding of local contexts and phenomenon under study [33]. AM and the research assistant were native speakers of the local language, discern the culture and health system, public health in profession, and skillful in qualitative research. The first author (FG) had a strong track record of research experience and publication on the epidemiology of tuberculosis in the setting. The authors (MD, AW, TG, RT and BS) were senior researchers and supervised the overall process of the study. None of the investigators was part of the TB control programme workforce in the setting, if were, could potentially convey biased reports to conceal apparent impairments due to health system deficiencies.

## Results

### Characteristics of respondents

A total of 41 respondents were interviewed for this study: 19 pastoralist patients delayed ≥2 months without seeking healthcare and 22 health workers at all levels of the TB control programme (Table 1).

**Table 1:** Characteristics of in-depth interview respondents in Somali Region, Ethiopia, 2018

Characteristics of respondents		
**Patient respondents (*n*** **= 19)**		**Number (%)**
Sex	Male	10 (52.6)
	Female	9 (47.4)
Age in years (median)		25–65 (36)
Literacy	Illiterate	15 (79.9)
	Read and write	4 (21.1)
Occupation	Pastoralist	16 (84.2)
	Agro-pastoralist	3 (15.8)
Marital status	Married	17 (89.4)
	Single	1 (5.3)
	Widowed	1 (5.3)
Family size (median)		3–12 (7)
**Health workers (*n*** **= 22)**		
Sex	Male	18 (81.8)
	Female	4 (18.2)
Age in years (median)		25–45 (28)
Educational status	Diploma	6 (27.3)
	First degree	15 (68.2)
	Master’s degree	1 (4.5)
Experience in TB Services (years)	2 to 5	13 (59.1)
	> 5	9 (40.9)
Responsibility	Regional level manager	2 (9.1)
	District level manager	4 (18.2)
	Healthcare providers	16 (72.7)

The results are presented under the two major themes: 1) Factors affecting TB services utilization, 2) Factors affecting effective service delivery. The themes are further subdivided into sub-themes and subsequent categories.

### Factors affecting TB services utilization

#### Knowledge and perceptions

##### Comprehensive TB knowledge

Patients had inadequate knowledge about TB cause, transmission modes, prevention methods and adverse disease outcomes. One patient only mentioned that TB is caused by a germ and spread by coughing, so majority of patients had not been taking to precautions to prevent household transmission. Also, we found patients who had never heard of TB before being diagnosed as TB patients. For instance, a 45 years old patient said:

“*I did not know about TB before. I have started learning about it after I was diagnosed with this disease.”*

##### Misconceptions/myth

Misconceptions/myths were identified regarding the cause and treatment of TB. All patients except one (who said ‘TB is caused by a germ’) misconceived that TB is acquired through exposure to cold air (predominantly stated), cold shower, workload, holding a heavy load, injury, curse, hereditary, sexual intercourse, and as emanated from other febrile illness. A 25 years old patient said:

*“I used to work as ration loader/daily laborer at WFP. There was cold pond water used to bath after the daily work. One day, I bathed there; I immediately felt the cold air penetrating my chest and got sick afterward.”*

Also, healthcare workers explained about a belief in the community that people believe TB drugs help to gain body weight. Higher body weight is seen as an indicative of wealth and beauty in the society, especially for women. This drives women to take TB drugs witout medical prescription. A district-level manager described:

“*Being overweight for women is a social dignity so that everyone wants to take TB drugs to gain weight without being diagnosed with TB.”*

##### Knowledge and perceptions of symptoms

Patients mentioned the various symptoms of PTB including cough, shortness of breath, chest pain, fatigue, and weight loss among others. However, some patients misconstrued TB as a disease with severe symptoms and associated symptoms like cough and shortness of breath with common cold or asthma. When patients were asked about initial perceptions of their illness, they replied, ‘*I thought it was common cold’; ‘I thought it was asthma’,* and *‘I thought it was not serious’*. The patients who perceived cough as a common symptom of the usual respiratory illnesses sought healthcare after the illness became severe. A 50 years old herder explained his experience:

“*I had been sick and had cough for months but never suspected TB. Instead, I thought I had asthma and was struggling to treat asthma. It was later on when I got weaker; I realized it was something serious...”*

##### Awareness of TB services

We encountered few patients who never heard about TB treatment and did not know it is provided for free so that they delay for days until getting money. Majorities of patients had never received health education about TB from healthcare providers and other channels, rather previously treated TB patients remain the main sources of TB information. Previously treated people also refer patients to seek healthcare. A 27 years old pastoralist said,

*“I had no idea about the cost. I assumed that the treatment would cost me 5,000 birr (Ethiopian currency; 1 USD = 32 birr), and I told my wife to sell cattle to get this money.….”*

The healthcare workers also affirmed that pastoralists have low awareness about TB services mentioning low or absence of health education services as the main contributing factors.

##### Perceived stigma

Negative perceptions including perceived discrimination and/or stigma mainly, and considering TB and HIV as embarrassment influenced the healthcare-seeking behavior of patients. Patients concealed their illness due to fear of discrimination. This is reaffirmed by DOTS providers who insisted that they encounter patients who refused to bring contact persons due to unwillingness to disclose their illness. A 45 years old patient said:

*“My family advised me several times to seek medical care before it became severe. Although I suspected that I had TB, I was denying it because I feared I would be discriminated.”*

#### Accessibility of TB services

It is further subcategorized into: accessibility (*distance to reach the nearest public health facility with/without TB services*); availability (*obtainability of TB services in a relatively nearest health facility*); affordability (*ability to pay any costs related to TB services including transport),* and acceptability (*TB services meet the expectations and demands of patients*).

##### Accessibility

Patients and healthcare workers underlined lack of nearby health facilities as one of the top barriers in the area. Patients travel several hours to reach the nearest health facilities. Beyond the distance to reach healthcare points, lack of road access and transportation to pastoral villages was stressed as terrible. Some zones have no access to public transport services that travel to district towns, let alone to *kebeles (lowest administrative unit in Ethiopian government structure)*. The limited access to healthcare services compelled patients to travel on foot for many hours and days, which constrained patients to delay in seeking healthcare and stay away from home in towns for long to complete TB treatments. An old herder described his journey as*,*

*“I spent one night in my way to here because I have to travel on foot a day and a night to arrive in Garbo where vehicles going to Jigjiga are found. The next day, I came to Jigjiga by vehicle.”*

An experienced HCP at a health center also said,

*“……We usually ask pastoralists to identify health centers in their area to link for treatment follow-up, but they stressfully claim there is nothing in their area. Due to this, we enforce them to stay here in the town for 6 months until the end of treatment.”*

##### Availability

Additional to the physical barriers, relatively accessible health posts and majorities of the health centers did not provide TB diagnosis and treatment services. Our interview with implementers confirmed that only less than half of the rural health centers had TB diagnosis and treatment services, and most of the health posts are nonfunctional in the region. Moreover, the health centers with diagnosis services were providing microscopic examination only and referring smear-negative patients to hospitals. A woman said,

*“We have a health center in Marsin district, but it does not have lab and TB drugs.”*

##### Affordability

TB medical services are provided free except few items such as X-rays. However, patients were incurred high costs for transportation, to buy food and rent houses in towns, and surplus costs in case of complicated illness. Patients with limited access to treatment in their area are forced to stay in towns until treatment is completed, but the living costs are unaffordable, and patients tend to discontinuetreatment follow-up. As a mitigation, DOTs providers handed over the full course of drugs required for 6 months regimen to patients to take home. A 40 years old pastoralist expressed his distress as,

*“I paid 500 birr (15.6 USD) for bus fare to arrive here and spent another 500 birr (15.6 USD) for meals and lodging. I have finished the money I had and I don’t know what to do next. I’m broken.”*

An HCP at a district hospital said,

*“…patients do not have a home in Degehabour town. They asked, ‘please give me all the drugs and let me go home?’ They do not afford to rent a house and buy food. So, what can we do? We have to give the drugs. If not, they will discontinue.”*

##### Acceptability

Patients expressed their complaints regarding the suitability of TB services including lack of services in residential areas, unaffordable cost to cover expenses in towns, long referral pathways, long waiting time, and multiple facility visits due to lack of services in frontline healthcare units. Due to lack of nearby services to their residence, patients were obliged to stay in towns to complete treatment, which causes work lay-off, financial bankruptcy, left out of family support, and loneliness and nostalgia in towns. A 30 years old woman said:

*“It is almost fourteen days since I have last seen my family. I have left little kids at home. I called them last night. When I said ‘Najib, how are you?’ my son cried. It is touching.”*

#### Pastoralism and climate change

During transhumance movements, patients struggle to access health services and discontinue treatment, and some health facilities were forced to close operations. Following seasonal movements to bushland areas during the dry season, treatment discontinuation and delay in healthcare-seeking were mentioned as common scenarios due to lack of access to healthcare in bush lands. During the fieldwork, we observed a significant decrement of patient flow in dry seasons (January to April) at health facilities in remote areas following relocation of the surrounding communities. A HCP at the health center said:

*“Pastoralists move from place to place searching for water and pasture for their cattle. They prefer their death to the death of their cattle. If a patient who took drugs for 15 days hears about rain in a certain place, the patient drops his treatment and moves. Then you will get that patient when he/she comes back after a long time.”*

Patients also repeatedly mentioned the influence of subsequent droughts on their livelihood and healthcare-seeking behavior. Frequent droughts in recent years destroyed livestock resources. Thus, patients had nothing to sell and get money to seek healthcare and feed their families. A nomadic pastoralist expressed the impact of drought as:

*“We had many cattle, but currently the drought has wiped out all of them. Previously it was easy to sell cattle and get money to cover costs of treatments for my family, but nowadays it is tough, that is why I came after begging money from other people.”*

#### Informal remedial practices

##### Traditional/religious healing practices

Patients and healthcare workers mentioned various informal healing practices including herbal medicines, spiritual healing practices and non-prescription medications. The practices attempted before visiting healthcare facilities were the use of herbs, *Khat* chewing as a pain reliever, religious healing (*Qur’an read by sheiks followed by something to drink*), visiting traditional healers, and self-medication with antibiotics and syrups. Patients who thought their illness was caused by a curse opted for religious leaders, traditional healers, or herbal remedies. The herbs used to treat pulmonary illnesses are locally named, *Galool (Acacia bussei), Liike, Ruman (Pomegranate), Boco (Calatropis procera), Mawo (Zigophyllum hilebrabdtii), and Tiire (Clerodedrum sp.)*. A 45 years pastoralist explained his experience as:

*“I didn’t think that I had TB. I used ‘Galool’ leaves, boiled like tea and drunk it. I then tried ‘Ruman’ leaves. At last, I became very weak and bed-ridden.”*

##### Medications by informal drug vendors

More than half of the interviewed patients sought first care at rural drug vendors where they received medications. Rural drug shops (mostly unlicensed) are common in remote areas of the region. Patients chose these drug vendors as first point-of-care due to their convenience and permissive process to purchase drugs and obtain treatments. At rural drug shops, patients are told as having ailments like pneumonia and anemia without medical examinations and given injectable and capsule medications. Patients only sought healthcare at a health center/hospital when the illness did not improve or got worse. A 27 years old patient said:

*“The first time I felt this illness, I went to the little pharmacy in our kebele. The vendor said ‘you have pneumonia’. He gave me injections, but I didn’t recover. Lastly, I decided to come here.”*

### Factors affecting effective service delivery

This theme describes the challenges that affect the provision and quality of TB medical services that are summarized into four sub-themes: (1) inadequate infrastructure; (2) inadequate resources; (3) health-system defects, and (4) cross-border movement.

#### Inadequate infrastructure

The providers and managers explained the limitations in basic infrastructure such as health facility designs, inadequate rooms, drug stores and water supply. The greater number of health centers in the region did not have adequate rooms for DOTs delivery and TB laboratory services due to design defects. We observed that the rural health centers have very small DOTs rooms (*roughly 2X3 square meters*) but no isolated TB laboratory, sputum collection and waiting areas, shelves for drugs, and water supply.. District level manager said:

*“There is no standard TB clinic in health centers. The majority of them have very narrow rooms that are not ventilated and isolated. We always shout for renovation, but……….”*

In addition to inadequacies in health centers, a DOTs provider at the referral hospital stressed the shortage of rooms for admitted and MDR patients. The DOTs center has been providing treatment follow-up services for MDR patients in the continuation phase, but with no separate room and shelf for MDR drugs. During our visit, we observed that MDR drugs were in plastic bags and put on an open bench in the DOTs room. The regional programme manager also stressed that the absence of MDR treatment center is unacceptable. The DOTs provider at the hospital said:

*“We have 1 room as TB ward and 1 room for DOTs. We admit all TB cases in this one room whether smear-positive or negative, male or female, new or retreatment. What is more dangerous is that MDR patients are linked to us for follow-up. Imagine, we are not trained, do not have the setup to care MDR patients or mask to protect ourselves.”*

#### Inadequate resources

##### Inadequate health workforce

Attrition and shortage of competent and motivated workers were manpower-related challenges that hindered the expansion and quality of TB services. Shortage of providers mainly laboratory professionals and programme experts at district health offices, high attrition and poor competencies at all levels were the main challenges. Majorities of rural health centers in the region did not have laboratory personnel and were staffed with junior and untrained providers. Moreover, the healthcare providers were less satisfied in their job due to inadequate salary, poor support, managerial malpractices, and lack of incentives and recognition. A DOTs provider with 10 years’ experience said:

*“I am bored. I have decided to quit my job next year. It is risky working in TB, but no one supports; no one motivates. Managers do not care about TB, but we are the ones who face the risks.”*

##### Inadequate supply (*consumables and equipment*)

The main shortfalls in supply management were interruptions of drugs and consumables (reagents, sputum cups, and masks), absence of Information Education Communication (IEC) aids and shortage of equipment. The Ethiopian Pharmaceutical Supply Agency (EPSA) is entitled to deliver drugs and consumables to service delivery points. However, drug stock-outs are common due to supply interruptions or delays from EPSA. A DOTs provider at a remote health center said:

*“There were cases we referred to Gode hospital since the drugs were finished. For example, we transferred one young patient very recently.”*

The regional laboratory is entitled to prepare and distribute reagents and other consumables for peripheral laboratories in the region, but none of the assessed laboratories were receiving supplies in recent times. Shortage of equipment and teaching aids were also common gaps. Only two hospitals possessed 4 Xpert MTB/RIF devices in the region. Nonetheless, a standard sample transfer system was not in place to utilize the existing facilities, and there was a complete absence of IEC materials of any kind at all levels of the healthcare tier. HCP at health center stated:

“*No IEC materials at all for years. I wish I had audiovisuals that would be opened every morning to teach patients in their Somali language.”*

##### Inadequacy of support (*supervision and funding*)

Inadequate supervision, budget and partner support were among the major challenges. The respondents at health facilities and district level claimed that supportive supervision was almost absent, and the regional level respondents, in turn, asserted that they were unable to reach all health facilities in the region due to structural, financial, logistic and manpower limitations. The absence of TB units at zonal and district levels, shortage of vehicles, and lack of road access to remote places were reported as the origins of supportive supervision gaps. The budget was another constraint, all activities were financed by Global Fund, but no direct government funding for TB programme. Nonetheless, there was no any partner organization supporting TB control programme in the area. A DOTs provider at district hospital said:

*“Support from regional health office? No! I don’t remember any supportive supervision that we have received except training.”*

#### Health system defects

##### Lack of zonal and district TB units

The health system structure lacks TB unit at zonal health department and district health office levels aligning administrative divisions. Due to the absence of zonal and district level TB units that should supervise the health facilities in their catchment, the regional coordination unit had to shoulder the supervision role for all health facilities in 11 zones and 96 districts of the region. The top manager described it as ‘unmanageable’ and one of the major bottlenecks. Also, lack of TB diagnosis in rural health centers, inefficiency of the health extension programme, and poor regulation of drug vendors were reported as deficiencies of the TB control programme that resulted from health system shortcomings. The top programme manager explained:

*“The problem I see is that the region’s healthcare system doesn’t have Zonal Health Department and TB focal persons at the district level. Due to this, the regional TB office has to supervise all health facilities in the region, which is not practically feasible”*

##### Poor engagement of private clinics

Private clinics were chosen as the first points-of-care by some patients, but most of the private clinics did not have TB services. As the evidence from RHB indicated, only three private clinics had TB diagnostic and treatment services in the region.

##### Lack of pastoralist-tailored approaches

The healthcare workers described the existing DOTs strategy as ‘unworkable for pastoralists’. The limited access to healthcare services turned out to be worse in dry seasons following the movement of pastoralists. The health system did not have any strategy in place that complements their healthcare demands during the seasonal movement. As a mitigation strategy to prevent defaulting, the DOTs providers offer the full course of drugs for patients to take home assuming they will continue taking their treatment wherever they are. However, the health system has no mechanism to evaluate treatment compliance and outcomes (whether cured, completed, died, or defaulted). A DOTs provider at a health center said:

*“The pastoralist communities do not have access to DOTs within walking distance. We urge them to stay in the town, but they can’t afford the living cost. Also, they have to stay away from their families, home and cattle. If you see from their side, it is difficult to comply with that. It will be better if a new approach can be found out.”*

#### Cross-border movement

Somali regional state of Ethiopia shares international borders with Djibouti, Somaliland, Somalia and Kenya. The study communities share similar ethnicity, language and religion with adjacent communities of neighboring countries. Interviews with patients and health workers unveiled that Ethiopian patients seek healthcare in neighbor countries or vice versa. For example, one patient said that three of his family members attended TB treatment in Somaliland. The healthcare providers claimed that they sometimes encounter patients that request referral to neighboring countries. However, the absence of a cross-border referral platform has been problematic to manage such cases. The manager at RHB described that there had been initiatives for cross-border collaboration years back, but it has not been materialized. A DOTs provider at a health center said:

*“….. A year before, one patient requested me to give him a referral paper to Somaliland. I said to him, ‘I can refer only to another health center/hospital in Ethiopia, but I do not have the mandate for an international referral.’ So, I tried to convince him to finish the treatment here. Unfortunately, he defaulted, and I could not trace him back.”*

## Discussion

**Fig. 1:**
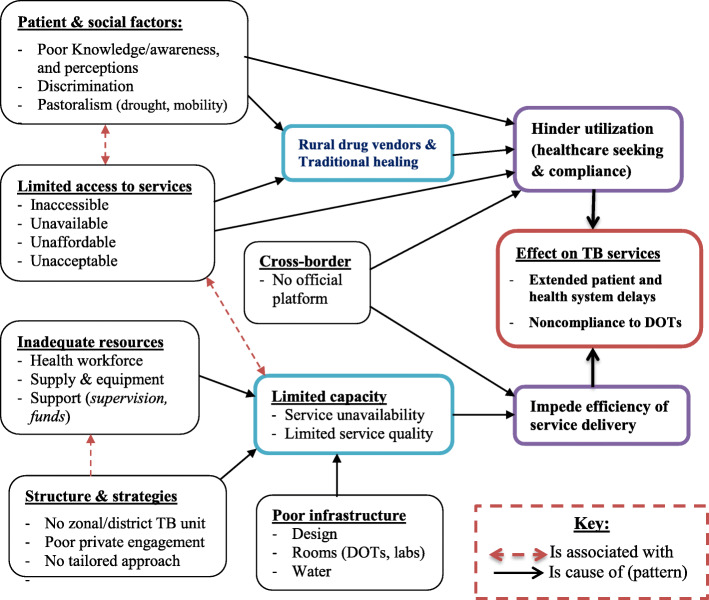
Interrelations and patterns of the barriers and challenges of TB services in pastoralist settings, Ethiopia

Through the eyes and experiences of patients, frontline providers and programme managers in eastern Ethiopia, we identified patient, societal context and health-system related challenges that affect the patients’ TB services uptake, and the healthcare system’s efficiency in provision of TB services for pastoralists. The major barriers affecting service utilization were poor knowledge, limited access to services, mobility, and informal medication practices; whereas, the major challenges affecting service provision included poor facility infrastructure, inadequate resources (*workforce, budget and supply*), lack of zonal health department and district TB unit, poor private engagement, lack of pastoralist-tailored approach, and cross-border movement. A framework for the interrelations and patterns of barriers and challenges of TB services in pastoralist communities was developed based on the concept of systems thinking [34, 35] (Fig. 1).

Our study reveals that poor TB knowledge, misconceptions, poor awareness of services and perceived discrimination were among the main factors affecting service utilization. The findings are consistent with reports from resource-limited settings [36–38]. The root cause of knowledge and awareness gaps was limited access to TB education and medical services. Community-based TB education was almost nonexistent, but previously treated TB patients were the main sources of information about TB. Although access to services without financial and geographical barriers is central to the End-TB strategy [39], pastoralists were not relishing the virtue of universal access as a result of thelack of nearby health facilities, or the absence of TB services at frontline facilities. This has been a prevailing challenge in pastoral areas of Ethiopia [40]. The inaccessibility worsens in the dry season when pastoralists migrate to remote areas. Hence, patients sought delayed healthcare and implementing DOTs has been perplexing. As mitigation, providers enforce patients to stay in towns to complete DOTs, but it is posing economic, psychological and social impacts such as excessive expenses for housing and food, lack of family support, nostalgia and work lay-off. Nonetheless, lack of socioeconomic support increases adverse treatment outcomes like a loss to follow-up that can result in drug resistance [41]. Thus, availing accessible and affordable TB services in primary healthcare units is a necessity to improve utilization of TB services in the setting [42, 43]. If cost-effective, provision of shelter and food services in health facilities can also improve DOTs compliance in pastoral areas [44].

Due to poor awareness and inaccessibility of services, pastoralist patients opted for traditional or informal remedies as initial points-of-care. Patients who perceived their illness as a common respiratory illness and associated with curse attempted herbal and religious healing practices, as shown in similar findings [45, 46]. What is unique in our finding is that most pastoralist patients commonly initiated first care at rural drug vendors who recklessly provide injectable and oral medications. Drug vendors are preferred due to their convenience and absence of health facilities in rural areas, so patients only sought healthcare after the medication attempts at drug vendors failed to improve their illness. This reveals that drug vendorsappeared to be the main barriers blocking patients’ healthcare-seeking to public health facilities. The inappropriate use of antibiotics by unauthorized and untrained drug vendors may result in undesirable disease outcomes, and an outbreak of resistance to the commonly used antibiotics may happen in the setting [47, 48]. Therefore, engaging rural drug vendors in presumptive case identification and referral is a unique opportunity to reduce delay and tackle the crisis of antimicrobial resistance in the setting.

Resource-related challenges affecting services’ delivery were inadequate infrastructure, manpower, supply, support and budget. Scarce resources hinder the availability and quality of healthcare services [15]. Due to shortage of healthcare providers mainly laboratory staff and poor infrastructure like lack of rooms and water supply, majorities of health centers in the region did not provide TB diagnosis and treatment services, which is incomparably lagging compared to elsewhere in Ethiopia [49–51]. Whereas, shortage of equipment, interruption of drug and reagent supplies, and lack of IEC aids were common incidents in health facilities with TB services, which could substantially hinder case detection and treatment efforts [49, 52]. For instance, only four Xpert MTB/RIF devices were available at the time of the study in two hospitals of the region although several hundred instruments have been deployed in Ethiopia to improve case detections and drug susceptibility testing [53]. Moreover, funding gaps, absence of partners/donors and expert limitations affected supervision activities. Providers’ job satisfaction which is essential to enhance TB programme performance [54] is also missing. This implies expanding services in existing primary healthcare units, employing healthcare workers with an attractive retention and capacity building strategy, ensuring persistent supply and local production of IEC materials are crucial to cascade the End-TB strategy targets on track in the setting. Efforts are also needed to look for potential partners that can fill the funding gaps.

Structural deficiencies restricted management capacities. The global strategy puts district-level TB units among the key elements of TB programme management to establish an enabling structure for proper workflow and management of activities [55]. However, the health-system structure lacks zonal and district TB units. As a result, the regional TB office was forced to shoulder all management activities in the region consisting of over 96 districts, but it was impossible. Decentralizing TB programme management into zonal and district levels should be done to create enabling structure for distribution of supplies, monitoring and evaluation, supervision, and support. Moreover, the poor engagement of private sector in TB services was another structural challenge. Private clinics and drug outlets are the first points of consultation for common illnesses in the study area, so failure to engage them - in identification, referral, diagnosis and treatment was a missed opportunity to capture missing TB cases, accelerate early detection and expand service coverage [48].

The lack of a tailored approach that complements pastoralists’ movements and the impacts of subsequent droughts was a critical setback. In the absence of a tailored approach that allows season-persistent access to healthcare services, the case finding and DOTs approaches cannot be successful but end up with unwanted outcomes specifically delayed healthcare seeking and treatment defaulting [10]. Characteristic service models such as mobile TB services and community engagement have been efficient and effective in hard-to-reach communities [56, 57]. Hence, mobile screening using the regional mobile health teams, one health approach and mobile DOTs through engaging community leaders can be useful strategies to enhance case finding and treatment adherence in pastoralist communities. The one health approach that integrates human-animal health services has been an effective and efficient mode of service delivery for pastoralists [58]. This study was conducted as part of one health approach in the setting and a related study has shown satisfactory results in community-based integrated disease surveillance, TB was not reported though. Hence, the one health approach can also help implement community-based TB case finding in the setting. Optional to mobile DOTs, novel technologies that assist patients’ drug intake at home will be very instrumental to mitigate the challenge of DOTs noncompliance.

Cross-border movement of pastoralists into/from neighboring countries (Somalia, Kenya and Djibouti) affected TB treatment compliance and management. A strategic framework for cross-border TB control programmeming was developed for highly mobile populations [59], but the initiative was not materialized to handle patients requesting cross-border referral that resulted in treatment discontinuations. Thus, the risk of drug resistance due to treatment discontinuation and exchange of resistant strains would be alarming among migratory patients in the setting [60]. This implies the need for founding cross-border collaboration platforms in the region.

As with any empirical investigation, this study is also subjected to certain limitations. Had we traced and interviewed defaulter patients, we would have provided a detailed understanding of the actual causes of defaulting in pastoralist setting directly from the voices of the patients facing the problem.

## Conclusion

TB control programme in pastoralist communities of Ethiopia has multifaceted challenges that are related to patient, pastoral/social context and health-systems. Poor knowledge, limited access to services, the pastoralist lifestyle/mobility, and rural drug retail practitioners are the major barriers affecting effective utilization of services; whereas, lack of zonal/district TB unit, inadequate resources, lack of pastoralist tailored approach, and cross-border movement are the major challenges affecting service provision. The combined effects of these shortfalls affect the provision, quality and utilization of TB medical services, and result in underperformance of the TB control programme in the pastoralist areas. Hence, in such setups, for the TB control programme to be successful, mobile screening and DOTs services, as well as engagement of rural drug vendors in presuming and referring cases will be instrumental to enhance early case finding and treatment compliance and to tackle drug resistance. Expanding TB services to primary healthcare units and decentralizing programme management will ultimately increase coverage and performance of the TB control programme.

## Supplementary Information

**Additional file 1.** Challenges in Delivery of Tuberculosis Services in Pastoralist Settings, Ethiopia: Clues for Reforming Service Models and Organizational Structures. Interview guide tools for delayed TB patients and TB care workers/managers.

## Data Availability

Direct quotes supporting the findings and conclusions are included within the article. The data contain confidential information, and consent has not been obtained for data sharing with identifiers. However, the data used and/or analyzed are available at the hands of the corresponding author and can be shared upon reasonable requests.

## Abbreviations

AFB: Acid Fast Bacilli
COREQ: Consolidated criteria for Reporting Qualitative research
DOTS: Directly Observed Therapy-Short Course
IEC: Information Education Communication
EPSA: Ethiopian Pharmaceutical Supply Agency
FMOH: Federal Ministry of Health
HCP: Health Care Provider
MDR: Multi-Drug Resistant
NTP: National TB Control Programme
PTB: Pulmonary Tuberculosis
RHB: Regional Health Bureau
TB: Tuberculosis
WHO: World Health Organization
WoHO: Woreda Health Office
USD: United States Dollar
