# Supplementary material for: Challenges in delivery of tuberculosis Services in Ethiopian Pastoralist Settings: clues for reforming service models and organizational structures
Source: BMC Health Serv Res. 2021 Jun 30;21:627. doi: 10.1186/s12913-021-06662-3 (PMC8246683; doi:10.1186/s12913-021-06662-3)
Supplement: Supplementary file 1 — Additional file 1. Challenges in Delivery of Tuberculosis Services in Pastoralist Settings, Ethiopia: Clues for Reforming Service Models and Organizational Structures. Interview guide tools for delayed TB patients and TB care workers/managers. [file 12913_2021_6662_MOESM1_ESM.docx]

**Challenges in Delivery of Tuberculosis Services in Ethiopian Pastoralist Settings: Clues for Reforming Service Models and Organizational Structures**

**Prepared by:**

- **Fentabil Getnet,**
- **Meaza Demissie,**
- **Alemayehu Worku,**
- **Tesfaye Gobena,**
- **Rea Tschopp,**
- **Alinoor Mohamed,**
- **Berhanu Seyoum**

This supplementary material contains the interview guide tools including consent acquisition used in this study entitled above.

## In-Depth Interview Guide Tool - TB Patients (age≥18 years)

## Participant Information Sheet and Informed Voluntary Consent Form

My name is _______________________________. I am working as a data collector for the study being conducted in this health facility led by Fentabil Getnet from Jigjig University and his colleagues from other institutions. I kindly request you to lend me your attention to explain you about the study and being selected as the study participants.

**Purpose/ aim of the study:** This study aims to explore the perception and practice of delayed TB patients prior to receiving TB medical care. It will provide deeper insights about knowledge of TB and its services, perceived barriers to prompt care seeking and remedies done at home to treat your illness and prevent transmission. This will inform policy makers, program implementers, clinical and practitioners to know the main barriers that hinder patients from seeking early diagnosis and treatment in your community.

**Procedure and duration:** I will ask you guiding question about the barriers that hinder you from early care seeking, the practices you did to treat your illness at home and household measures you took to prevent transmission before you obtain medical care. Then you will respond and discuss the questions accordingly. I will take notes to write what you say. However, I will not be able to write all what you say. Therefore, I will use this audio recorder (***show the device***) in order to capture all the information that I may miss during hand writing. Then will listen to audio later on and transcribe to my computer. So I kindly request you to grant me the time and cooperation for data collection.

**Risks and benefits:** The risk of being participating in this study is very minimal; and there will not be anything that could impose harm (physical, social, emotional, mental, political, work and religious). There would not be any direct payment for participating in this study. But the findings from this research may reveal important information for the local health planners and your doctors for future actions.

**Confidentiality:** The information you will provide us will be confidential. There will be no information that will identify you in particular. The findings of the study will be general for the study community and will not reflect anything particular of individual persons or housings. No reference will be made in oral or written reports that could link participants to the research.

**Rights*:*** participation in this study is fully voluntary. You have the right to declare to participate or not in this study. If you decide to participate, you have the right to withdraw from the study at any time and this will not label you for any loss of benefits which you otherwise are entitled. You do not have to answer any question that you do not want to answer.

***Contact address:*** if there are any questions or enquires anytime about the study or the procedures please contact:

| **Address of Principal Investigator**  Fentabil Getnet  Jigjiga University, COHMS  Mobile: +251 913 289 380  P.O.Box: 1020, Jigjiga, Ethiopia  Email: [b.infen4ever@gmail.com](mailto:b.infen4ever@gmail.com) | **Address of IHRERC: HU,CHMS**  **Office:** Tel No: +251 254 660 708  P.O.Box: 235, Harar, Ethiopia | **Address of AHRI/ALRT Ethical Review Committee**  Tel No.: +251 113 481 289  Addis Ababa, Ethiopia |
| --- | --- | --- |

***Declaration of informed voluntary consent:*** I have read the information sheet or it was read to me. I have clearly understood the purpose of the research, the procedures, the risks and benefits, issues of confidentiality, rights and the contact addresses for any queries. I have been given the opportunity to ask questions for things that may have been unclear. I was informed that I have the right to withdraw from the study at any time or not to answer any question that I do not want. Therefore, I declare my voluntary consent to participate in this study with my initials and fingerprint or signature.

Name and signature of participant: ____________________________________________

Name and signature of data collector: ______________________________________________

**Witness** (in case the participant is illiterate)

Name and signature of witness: _________________________________________

Date: ________________________________________

## Questions for Delayed PTB Patients

IDI Guiding Questions on perception and practice of Delayed Patients on TB Prevention and Treatment

**Respondent Background Information**

1. Respondent Profile

Sex: ________Age: _____; Educational status_______________; Religion_______________

Marital status_________ role in family ________ family size_______ Occupation____________

1. **Name of Interviewer:**_________________________ Signature: _______

Date of Interview: __________Time started: ______Time ended: ________

1. **Knowledge of Tuberculosis**
2. What do you know about your current illness? **Probe**: the name of the disease? Its dangerousness? The consequences?
3. Can you explain me what you know about tuberculosis disease? **Probe**: the disease, the body parts affected, the cause, who are more susceptible, the dangerousness of the disease?
4. What are the major signs and symptoms that a person who is infected with TB manifested? Can you distinguish a TB patient by observing the ill condition? How?
5. How does someone get tuberculosis? **Probe**: exposure to cold air, evil curse, association with HIV, transmitted from infected person?
6. Is tuberculosis preventable? If yes, how could it be prevented?
7. Where did you get information about tuberculosis?
8. Have you ever obtained health education on tuberculosis? When and who provided it? What were the contents?
9. **Awareness and accessibility of TB Services**
10. Why did you go to the doctor when you developed pulmonary illness?
11. Did you go to another place before this hospital? *If yes, where did you go? and why*?
12. Why did you come to this hospital? Who told you to come here?
13. What do you think is a hospital good for what you have, what do they do to help you?
14. What did the doctors do to you? What did they give to you?
15. For how long do you have to take the tablets? How do you get these tablets?
16. What did you know about these tablets and the diagnosis of TB before? **Probe**: *how diagnosed? How treated and for how long it takes?*
17. Where else can someone get the treatment services for TB? Could you get TB treatment services nearby to your home? If yes, why did you prefer this hospital to the nearest one? **Probe**: *the type of the nearest facility, the services available…*

How do you come to the facility, how long does it take you to get there, how much does it cost you? **Probe**: *total time it takes to arrive (hours or days)*? *Walking distance (in time)? By vehicle and its cost?*

1. How do you see the accessibility of TB services to your village? Can everyone in your family or village including children, women and elders easily access the TB services at the health facility?

Can you access the any health facility all the year, what are the challenges? Are there seasons when you cannot get health facilities? ***Probe:*** *seasonal variability to access the service?*

How did you get the service at the health facility? What do you like about the service? What do you dislike? **Probe**: *the quality of the services on the perspective of the respondents*?

1. Have you ever heard of the services provided at this hospital before? What about TB diagnosis and treatment services?
2. What did you know about the service charges before? How much did you cost to get TB diagnosis and treatment services? Is it affordable to you or your family?
3. How many days have you stayed away from your home or work in order to get TB diagnosis and treatment services? **Probe**: *how much does it impact their usual or family life*?
4. Where did you get information about tuberculosis services?
5. **Reasons of delayed care seeking:**
6. You have been sick for two or more months? Why did you stay till now without coming to the hospital? Can you tell me the main reasons? **Probe**: money, access, other traditional treatments, illness not too serious…. please ask more explanations for each reason the respondent mentions?
7. Who decides to seek care if someone gets sick in your family? **Probe**: *husband, wife*?
8. Can women in your family or village decide to go to hospital when they or their children get ill? **Probe**: *check the influence of men on women’s or children health care demanding*
9. You have been diagnosed with TB now, are you affected by knowing you have TB? How will this change your life? What did your family say? **Probe**: *fear of* *social stigma/discrimination to TB*
10. When you got this illness, did something or someone hinder you from going to the doctor/hospital?
11. How do you feel about going to a doctor? How did the medical staffs treat you? What did they do to you? Do you know why they do these things for you?
12. Would you recommend a friend or a family member who is sick to come to the hospital? Why? **Probe**: check their perception to medical system or medical staffs?
13. What was your feeling to hospitals or doctors before you got treatment? **Probe:** *if the patient had perception, ask why and did it hinder him/her from going to hospital?*
14. **Household action to illness**
15. What did you do when you had initially developed pulmonary illness? Who urged you to do so? **Probe**: *family, friend, neighbor…?*
16. Did you try home remedies? If yes, traditional medicines or modern medicines? **Probe**: ask for more clarification of the types of remedies, dose, and administration?
17. Do you or others in your community purchase TB drugs from drug vendors or shops without physician prescription? (*N.B: note for interviewer: it is to assess TB drug black market*)
18. Had you visited traditional healers or religious leader for you current illness? If yes, what did you obtain as healing practice from the healer/religious leader? **Probe**: any medicine (herbal, modern …), pray………
19. **Household measures to prevent transmission**
20. When you were initially ill, were you afraid that other family members would get the same thing as you? Why?
21. *(If yes to the above question)*, what actions you did to avoid contamination your family? **Probe**: *Actions at home before treatment*?
22. Could you explain me your house conditions? Type (traditional nut or metal roof), Number of rooms, presence of windows, ventilation condition?
23. How could you think the medical service help the community better? Do you have any additional ideas, comments, suggestions?

## Key Informant Interview Guide Tool- TB service providers/managers

## Information Sheet and Informed Consent Form for Healthcare providers

My name is _______________________________. I am working as a data collector for the study being conducted in this health facility led by Fentabil Getnet from Jigjig University and his colleagues from other institutions. I kindly request you to lend me your attention to explain you about the study and being selected as the study participants.

**Purpose/ aim of the study:** This part of the study seeks to provide deeper insights into the challenges and barriers the health system and particularly you the frontline health care providers confront while providing TB services to pastoralists. The challenges and barriers could be related to health management system, logistic and supply management, provider proficiency, patient/societal (care seeking & compliance to care), and infrastructure factors. Thus, this study will put in valuable information to challenges of the health system in the provision of these TB services to pastoralists of Ethiopian Somali Regional State.

**Procedure and duration:** I will ask you guiding questions about TB services provided by your working unit, challenges and barriers usually confronted by you and hamper your service provision activities. Then you will respond and discuss the questions accordingly. I will take notes to write what you say. However, I will not be able to write all what you say. Therefore, I will use this audio recorder (***show the device***) in order to capture all the information that I will miss during hand write. Then will listen to audio later on and transcribe to my computer. So I kindly request you to grant me the time and cooperation for data collection.

**Risks and benefits:** The risk of being participating in this study is very minimal; and there will not be anything that could impose harm (physical, social, emotional, mental, political, work and religious). There would not be any direct payment for participating in this study. But the findings from this research may reveal important information for the healthcare system for future actions.

**Confidentiality:** The information you will provide us will be confidential. There will be no information that will identify you in particular. The findings of the study will be general for the study community and will not reflect anything particular of individual persons or housings. No reference will be made in oral or written reports that could link participants to the research.

**Rights*:*** participation in this study is fully voluntary. You have the right to declare to participate or not in this study. If you decide to participate, you have the right to withdraw from the study at any time and this will not label you for any loss of benefits which you otherwise are entitled. You do not have to answer any question that you do not want to answer.

***Contact address:*** if there are any questions or enquires anytime about the study or the procedures please contact:

| **Address of Principal Investigator**  Fentabil Getnet  Jigjiga University, COHMS  Mobile: +251 913 289 380  P.O.Box: 1020, Jigjiga, Ethiopia  Email: [b.infen4ever@gmail.com](mailto:b.infen4ever@gmail.com) | **Address of IHRERC: HU,CHMS**  **Office:** Tel No: +251 254 660 708  P.O.Box: 235, Harar, Ethiopia | **Address of AHRI/ALRT Ethical Review Committee**  Tel No.: +251 113 481 289  P.O.Box: ___Addis Ababa, Ethiopia |
| --- | --- | --- |

***Declaration of informed voluntary consent:*** I have read the information sheet or it was read to me. I have clearly understood the purpose of the research, the procedures, the risks and benefits, issues of confidentiality, rights and the contact addresses for any queries. I have been given the opportunity to ask questions for things that may have been unclear. I was informed that I have the right to withdraw from the study at any time or not to answer any question that I do not want. Therefore, I declare my voluntary consent to participate in this study with my initials and fingerprint or signature.

Name and signature of participant: ____________________________________________

Name and signature of data collector: __________________________________________

Date: _____________________________

## Questions for Key informants (TB care provider and program managers)

**Background information**

**Respondent Profile**

Sex: ________________ Age: _________; Educational status & profession_______________;

Experience_______________; Experience in TB services____________

**Name of Interviewer:**_________________________

Signature: _______ Date of Interview: __________Time started: ______Time ended: ________

**Questions**:

1. Could you explain the TB services your office/working unit provides? What are the specific activities? **Probe**: program management? Health education? Suspect identification and referral? Vaccination? Diagnosis? Treatment? Follow up?
2. How do you express your experience in the provision of TB services? What are the good deeds and main challenges you have faced so far?
3. How do you rank the support of RHB, Woreda Health Offices, NGOs or other stakeholders on the services you provide regarding TB? What are the challenges of TB services provision related to the health management system/structure?
4. How do you see the accessibility and availability of TB services in this pastoral community?
5. What are the barriers and challenges of TB services provision related to facility infrastructure (labs, TB wards…)?
6. What are the barriers and challenges you have confronted that are related to logistics and supply management in the provision of TB services? **Probe**: IEC, drugs, lab reagents, equipment?
7. What do you think are the challenges related to TB care providers’ competency, skill, training and commitment?
8. How do you relate pastoralism with Tuberculosis? **Probe**: anything special to them, their vulnerability, access to services?
9. Does pastoral way of life of the community affect the provision of TB services? If so, how and why?
10. How is the health care seeking behavior of pastoralists to utilize TB services?
11. How do you see the mobile nature of pastoralists and compliance to TB treatment? What is your experience regarding the compliance/adherence of pastoralist TB patients to TB DOTS?
12. From your experience, how do you see the effect of migration on TB treatment matters like compliance? (**Probe**: including cross-border movement between ESRS & Somaliland or Somalia; refugees; internally displaced people)
13. How do you see the suitability of the current DOTS program for mobile pastoralists?
14. Are there drug vendors or shops or individuals who sell TB drugs without prescription of TB care providers? (***Probe****: TB drugs’ black marketing, contraband…)*. If yes, would you tell me about the trade network including the source of drugs, sellers, regulatory activities…?
15. What do you think is a better approach to provide TB medical services for pastoralists effectively?
16. Any additional ideas, comments, suggestions that could help to improve TB services provided to the community?

## Questions on Care seeking practice of patients- TB Care providers

It is intended to collect further information on the patients’ perception and practice of TB care from TB care providers’ perspective.

1. In your practice, what are the common perceptions (misconception, myth) regarding tuberculosis that you usually encounter in this community? Probe: belief on the causality, transmission, treatment ……..
2. What are the common traditional or home remedies used by the pastoralists to treat tuberculosis? (probe: TB drug black marketing)
3. How do you describe the medical care seeking behavior of the pastoralists from the perspective of your experience?
4. What do you think are the main factors/reasons for delayed care seeking in this pastoralist community?
5. How do you notice the accessibility and availability of tuberculosis services for pastoralists?
6. How do you see the compliance of the pastoralist patients to tuberculosis medical services?
7. Any additional ideas, comments, suggestions that could help to improve TB services provided to the community?
